# Relative importance of intensity and spectrum of artificial light at night in disrupting behavior of a nocturnal rodent

**DOI:** 10.1242/jeb.247235

**Published:** 2024-07-15

**Authors:** Travis Longcore, Sophia Anne Marie B. Villanueva, Kyle Nguyen-Ngo, Cristina A. Ghiani, Benjamin Harrison, Christopher S. Colwell

**Affiliations:** ^1^ UCLA Institute of the Environment and Sustainability, 619 Charles E. Young Drive East, La Kretz Hall, Suite 300, Box 951496, Los Angeles, CA 90095-1496, USA; ^2^ UCLA Department of Integrative Biology and Physiology, 612 Charles E. Young Drive East, Box 957246, Los Angeles, CA 90095-7246, USA; ^3^ UCLA Semel Institute for Neuroscience and Human Behavior, Department of Psychiatry and Biobehavioral Sciences, 760 Westwood Plaza, Los Angeles, CA 90095, USA; ^4^ UCLA Department of Pathology and Laboratory Medicine, 10833 Le Conte Avenue, Los Angeles, CA 90095-1732, USA; ^5^ Korrus, Inc., 837 North Spring Street, Suite 103, Los Angeles, CA 90012, USA

**Keywords:** Activity, Masking, Entrainment, Correlated color temperature, Spectral sensitivity, *Mus musculus*

## Abstract

The influence of light spectral properties on circadian rhythms is of substantial interest to laboratory-based investigation of the circadian system and to field-based understanding of the effects of artificial light at night. The trade-offs between intensity and spectrum regarding masking behaviors are largely unknown, even for well-studied organisms. We used a custom LED illumination system to document the response of wild-type house mice (*Mus musculus*) to 1-h nocturnal exposure of all combinations of four intensity levels (0.01, 0.5, 5 and 50 lx) and three correlated color temperatures (CCT; 1750, 1950 and 3000 K). Higher intensities of light (50 lx) suppressed cage activity substantially, and consistently more for the higher CCT light (91% for 3000 K, 53% for 1750 K). At the lowest intensity (0.01 lx), mean activity was increased, with the greatest increases for the lowest CCT (12.3% increase at 1750 K, 3% increase at 3000 K). Multiple linear regression confirmed the influence of both CCT and intensity on changes in activity, with the scaled effect size of intensity 3.6 times greater than that of CCT. Activity suppression was significantly lower for male than for female mice. Assessment of light-evoked cFos expression in the suprachiasmatic nucleus at 50 lx showed no significant difference between high and low CCT exposure. The significant differences by spectral composition illustrate a need to account for light spectrum in circadian studies of behavior, and confirm that spectral controls can mitigate some, but certainly not all, of the effects of light pollution on species in the wild.

## Teaser

**Editors' choice:** Light intensity and spectrum interact to determine influence on locomotor activity of a nocturnal rodent, with amber light having half the effect of full-spectrum light at 50 lx.

## INTRODUCTION

The fields of chronobiology, with its focus on physiological responses to daily cycles, and conservation biology, in its attention to the question of nighttime light pollution, are beginning to converge (Aulsebrook et al., 2020; Dominoni, 2015; Dominoni and Nelson, 2018; Grubisic et al., 2019; Hölker et al., 2021). Researchers have investigated patterns of sleep, hormone production and activity patterns in model organisms for decades in the laboratory and increasingly now with wild organisms (Aulsebrook et al., 2018, 2020; Rattenborg et al., 2017; Robert et al., 2015). The influences of artificial light at night in the wild have long been documented for foraging (Frey, 1993; Goertz et al., 1980; Kotler, 1984; Rohweder and Baverstock, 1996; Sick and Teixeira, 1981), activity patterns (Barber-Meyer, 2007) and reproduction (Rawson, 1923; Rowan, 1938) across a range of taxonomic groups. Research is now linking laboratory and field-based studies to describe the mechanisms and thresholds associated with these influences (de Jong et al., 2015; Dominoni et al., 2019; Schirmer et al., 2019; Simons et al., 2022).

Responses to light stimuli fall into two related categories: influences on circadian rhythms themselves, which involve the entrainment of an endogenous oscillator, and changes directly in the response to an external signal without influencing the endogenous oscillator, known as masking (Mrosovsky, 1999). In both instances, photons are detected by intrinsically photosensitive retinal ganglion cells (ipRGCs) that express the photopigment melanopsin, which has a peak sensitivity to light at 480 nm (Hattar et al., 2002; Panda et al., 2005). Rod and cone photoreceptors also provide inputs to ipRGCs (Altimus et al., 2008; Dacey et al., 2005; Hattar et al., 2002; Lall et al., 2010; Lucas et al., 2014; van Diepen et al., 2021), indicating that the circadian system is sensitive to a broad spectrum of light (Foster et al., 2020). The light induction of the immediate early gene cFos in the suprachiasmatic nucleus (SCN) is a reliable measure of photic regulation of the circadian clock (Caputto and Guido, 2000; Rieux et al., 2002; Wang et al., 2023). Researchers of the ecological implications of artificial light on free-roaming wildlife are concerned with circadian entrainment and associated daily rhythms in behavior and physiology (Grubisic et al., 2019) and also with masking behaviors. Such masking behaviors are not always identified by this name in the ecology and conservation literature, but rather as behavioral ‘disruptions’ or exploitation of the ‘night light niche’ (Schwartz and Henderson, 1991).

Assessment and mitigation of the effects of nighttime light exposure has focused on spectrum, because of the characteristic pattern of the melatonin suppression curve seen in humans (Brainard et al., 2001). Manipulating spectral composition of light is a means to influence circadian and seasonal physiological responses, in both humans (Brainard et al., 2015; Nagare et al., 2019; Rea et al., 2010; Souman et al., 2018) and wildlife (Dimovski and Robert, 2018). Avenues of influence on nocturnal behaviors need not be limited to the circadian system, with the differing visual systems of taxonomic groups allowing for behavioral responses (masking) that are influenced by the spectral composition of light. For example, many insects see in the ultraviolet spectrum, and they generally exhibit positive phototaxis to light in this portion of the spectrum (Cleve, 1964; Donners et al., 2018), whereas humans do not perceive these wavelengths of light. These behavioral responses have already been the subject of proposals to mitigate effects of light pollution (Longcore et al., 2015, 2018; Rodríguez et al., 2017; van Grunsven et al., 2014). Although researchers agree that both light intensity and spectrum are important to wildlife (Davies et al., 2013; Gaston et al., 2012; Longcore and Rich, 2017), their relative importance deserves more investigation, such as has been done to some degree in laboratory studies with model organisms. Furthermore, the influence of naturalistic levels of light at night, which fall in the range of conditions experienced in nature without light pollution, deserve far more investigation, both for laboratory studies focusing on mechanisms (Walbeek et al., 2021) and to investigate natural responses to these conditions in wild organisms as a baseline to understand light pollution, even if that pollution may ‘only’ increase illumination from that similar to a quarter moon (0.01 lx) to that of a full moon (∼0.1–0.3 lx) (Brown, 1952; Kyba et al., 2017). Typical laboratory chronobiological studies investigating entrainment with model organisms consider 5 lx to be ‘dim’ light (Walbeek et al., 2021), and this is also the typically cited minimum illumination that causes melatonin suppression in humans, notwithstanding broad intraspecific variability (Phillips et al., 2019).

Some barriers inhibit studies that fully explore the effects of light at night at naturalistic intensities and at different spectral compositions in the laboratory and field. In the field, it is difficult to deliver a constant and known dose of light to species, given existing light pollution at study sites and technological limitations of both lights that might be deployed and the equipment to measure both intensity and spectrum at naturalistic (<0.3 lx) conditions. Rather, studies often use distance from brighter lights of known spectral composition to create gradients that then decrease to the ambient conditions (Shier et al., 2020). Laboratory studies often keep model organisms (mice and rats) in near-total darkness as the nighttime control, even though this condition is highly unnatural and outside the range of natural conditions in which these species evolved (Aulsebrook et al., 2022). Logistical constraints on providing light of specified spectral content and at naturalistic levels must be overcome to investigate and improve understanding of the interaction between spectrum and intensity in the influence of light on both behavioral and physiological responses in animals.

Custom laboratory lighting systems using light-emitting diodes (LEDs) now allow for delivery of specific spectral outputs to the same organisms over time and at different intensities. Such systems can deliver different color temperature light using combinations of individual colors of LEDs making up an array. Through use of dimming and neutral density filters these spectral compositions can be delivered across a range of ecologically relevant intensities. Field studies of wildlife habitats using new measurement tools, such as calibrated cameras collecting hemispherical images (Jechow et al., 2017; Pendoley et al., 2012; Simons et al., 2020) can define naturalistic conditions for species and habitats of interest.

In this study, we evaluated combinations of intensity and spectral composition of light in controlled conditions for their impact on a behavioral response in the house mouse, *Mus musculus*. The lowest light levels used are comparable to measurements taken in open desert habitat that is home to many nocturnal rodent species. The house mouse is nocturnal, and despite long use in laboratory settings, retains its strongly suppressed behavior under light at night (Busch and Burroni, 2015). Not all rodents have a similar response, but many do show a strong moonlight/artificial light at night aversion (Prugh and Golden, 2014). Therefore, we used the house mouse in a laboratory setting to investigate the interactions between intensity and spectrum on nighttime activity and circadian entrainment.

Spectral response curves for circadian regulation and visual responses are known for mice (Jacobs and Williams, 2007; Rocha et al., 2016). Based on preliminary calculations with these response curves and the spectral power distributions of the proposed light sources based on Longcore et al. (2018), we hypothesized that: (1) lower correlated color temperature (CCT) lights would deviate less from control conditions because they overlap less with the mouse visual and melanopic responses, (2) illumination similar to the darker half of the lunar cycle would result in greater activity compared with much darker conditions regularly created in laboratory conditions (see also Walbeek et al., 2021) and (3) indicators of circadian regulation, such as light induction of cFos in the SCN, would be less impacted by the lower CCT light in comparison to the higher CCT light delivered at equal apparent intensity. The study involved nocturnal exposure of laboratory wild-type (WT) mice to a pulse of light of varying intensity and color temperature and evaluation of the changes in its cage activity elicited by these different light treatments.

## MATERIALS AND METHODS

### Light treatments

To calibrate the dimmest exposure, field data were taken in Coachella Valley, CA, USA, along State Route 62 as an example of open desert habitat with many native rodent species, located >3 km from the nearest urban development. Data were collected using a Sky Quality Camera (Euromix Ltd, Llubljana, Slovenia) on nights with a new moon and after astronomical twilight. Cloud cover, which reflects light and increases light pollution, was variable. Both cosine-adjusted illuminance and hemispherical illuminance were extracted from the imagery. We plotted the relationship between hemispherical illuminance and cos-adjusted illuminance because hemispherical illuminance is important to exposure (light from all directions) whereas light meters used to measure light in laboratory conditions measure cos-adjusted illuminance. The average scalar illuminance at 15 locations from 10 to 400 m from a highway was 0.020 lx. This corresponded to a cos-adjusted illuminance of 0.007 lx. These compare with the illumination produced by a quarter (crescent) moon at its brightest of 0.008 lx (Krisciunas and Schaefer, 1991). The lower limit of our light meter is 0.01 lx, so we set this as the lowest exposure for the experiment so that we could measure it accurately. At the upper end, we chose both 5 lx, which is known to affect rodent circadian rhythms and could be experienced by rodents near roadway lighting, and 50 lx, well above known impact thresholds, to see the maximum influence of spectral differences. Between these extremes we selected 0.5 lx, which is somewhat greater than the light of a full moon (Kyba et al., 2017).

**Fig. 1.**
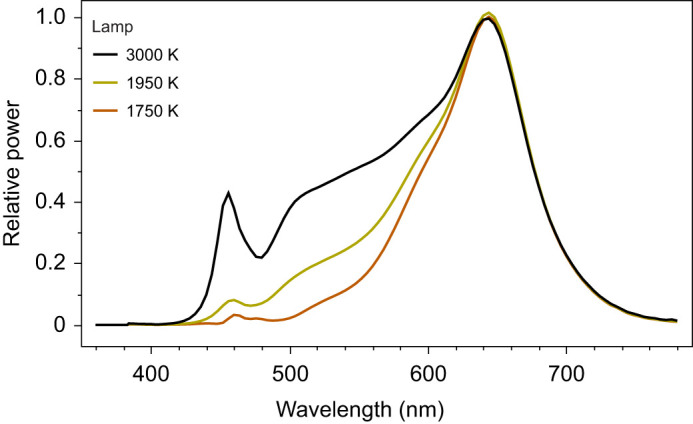
Spectral power distribution of lights used in experiment of 1750, 1950 and 3000 K correlated color temperature (CCT) (Korrus, Inc., Los Angeles, CA, USA).

Custom lighting systems using LEDs were obtained from Korrus, Inc. (Los Angeles, CA, USA). The systems could be adjusted to spectral output to achieve different color temperatures. Because any particular CCT can be achieved in different ways, we compared possible outputs against known lamp types in terms of their predicted melanopic effect, using methods described in Longcore et al. (2018). We selected three configurations to represent a range of melanopic effects when compared with daylight (D65) and that fall within the range of commercially available outdoor lighting. Ordered from highest melanopic effect to lowest compared with D65, they were 3000 K (54%), 1950 K (30%) and 1750 K (13%) (Fig. 1). For comparison, the melanopic effect as a percent of D65 for typical lamps is as follows: 4200 K LED streetlight (56%), 3000 K LED streetlight (45%), high pressure sodium streetlight (18%) and phosphor-coated amber LED streetlight (10%).

### Study organism

This study was performed in strict accordance with the recommendations in the Guide for the Care and Use of Laboratory Animals of the National Institutes of Health. All animals were handled according to approved institutional animal care and use committee (IACUC) protocols of the University of California Los Angeles.

Sixteen WT mice (*Mus musculus* Linnaeus 1758) (C57Bl/6J https://www.jax.org/strain/000664; 8 males, 8 females) were obtained at 3 months of age from Jackson Laboratory and placed into environmental control chambers. The sample size per group was determined by both our empirical experience with the variability in the prior measures in mice and a power analysis (SigmaPlot, SYSTAT Software, San Jose, CA, USA) that assumed a power of 0.8 and an alpha of 0.05. Although this strain of mouse does not produce melatonin, it has melatonin receptors (Ebihara et al., 1986) and its circadian system (light detection, physiological, and molecular mechanisms) is extremely well understood. The experiments were ended when the mice were 6 months old. Each mouse was individually housed to obtain rhythms in activity based on wheel running activity. The environmental chambers control sound, maintain temperature and humidity, and allow the light to be varied in intensity and spectral properties using the custom LED illumination system.

### Photic suppression of nocturnal activity (negative light masking)

Mice were entrained to a light:dark cycle consisting of 12 h of light (350 lx; 3000 K) and 12 h of dark for at least 2 weeks, and then exposed to four levels of light intensity (0.01, 0.5, 5 and 50 lx), each at three different CCTs (1750, 1950 and 3000 K) for 1 h at Zeitgeber time (ZT) 14, e.g. 2 h after lights off (ZT0, lights on; ZT12, lights off). Mice were tested once per week with at least 6 days of recovery in 12 h:12 h light:dark conditions between exposures. Each mouse was subjected to all 12 light intensities in the following order: 5, 0.5, 0.01 and 50 lx. For each intensity, the order of CCT exposure was 3000, 1950 and 1750 K.

Home cage activity based on wheel running behavior was reported to a VitalView data recording system (Mini Mitter, Bend, OR, USA). Wheel rotations were recorded in 3 min bins, and analysis was carried out using the El Temps chronobiology program (A. Diez-Noguera, Barcelona, Spain; http://www.el-temps.com/principal.html). Locomotor activity level during the light exposure was compared with the activity at the same phase (ZT14–15) in the prior day. The percent change in activity was then calculated.

To analyze the data, we first compared suppression in activity using CCT and illuminance both as categorical variables and testing for significant differences for each while holding the other constant (Tukey–Kramer HSD for multiple comparisons). Then, to account for potential interactions between CCT and illuminance, we built a generalized linear model (Nelder and Wedderburn, 1972) with a normal distribution and log link function using suppression as the dependent variable and CCT (categorical), illuminance (continuous) and CCT×illuminance as the independent variables. Sex and mouse ID were included in separate models as additional explanatory factors and models were compared using the Akaike information criterion corrected for small sample size (AICc). Effect sizes (the relative contributions of each variable) were compared by centering each factor at the mean and scaling by half of its range. All statistical tests were performed using JMP Pro 17 (SAS Inc., Cary, NC, USA).

### Photic induction of cFos in the SCN

A separate cohort of male and female WT mice (3–4 months old; *n*=3 per sex) was housed in normal light:dark conditions and exposed to either 1950 or 3000 K light (50 lx) for 1 h at ZT14. The animals were euthanized with isoflurane (30–32%) at ZT15 and transcardially perfused with phosphate-buffered saline (PBS, 0.1 mol l^−1^, pH 7.4) containing 4% (w/v) paraformaldehyde (PFA, Sigma-Aldrich). The brains were rapidly dissected out, post-fixed overnight in 4% PFA at 4°C, and cryoprotected in 15% sucrose. Coronal sections (50 μm) containing the middle SCN were obtained using a cryostat (Leica, Buffalo Grove, IL, USA), collected sequentially, and paired along the anterior–posterior axis before further processing. Immunofluorescence was performed as previously described (Lee et al., 2018; Wang et al., 2023). Briefly, free-floating coronal sections containing the mid-SCN were blocked for 1 h at room temperature (1% BSA, 0.3% Triton X-100, 10% normal donkey serum in 1× PBS) and then incubated overnight at 4°C with a rabbit polyclonal antiserum against cFos (1:1000, clone 9F6, Cell Signaling) followed by a Cy3-conjugated donkey-anti-rabbit secondary antibody (1:300, Jackson ImmunoResearch Laboratories, Bar Harbor, ME, USA). Sections were mounted and coverslips applied with Vectashield mounting medium containing the nuclear staining DAPI (4′-6-diamidino-2-phenylinodole; Vector Laboratories, Burlingame, CA, USA), and visualized on a Zeiss AxioImager M2 microscope (Zeiss, Thornwood, NY, USA) equipped with an AxioCam MRm and a motorized stage.

### cFos positive cell counting

Z-stack images (7 μm interval, 15 images) of both the left and right middle SCN were acquired with a 20× objective using the Zeiss Zen digital imaging software. Three observers masked to the experimental groups performed the cell counting. The SCN was visualized using the DAPI nuclear staining, traced, and the cells immuno-positive for cFos were counted with the aid of the Zen software tool ‘marker’ in three to five consecutive sections. The values obtained in the left and right SCN of each slice were averaged. The means of the three to five slices were then averaged to obtain one value per animal and are presented as the mean±s.d. of six animals per light treatment. Data analysis was performed using Prism (Version 9.5.0; GraphPad Software, La Jolla, CA, USA). The data passed the Shapiro–Wilk and Kolmogorov–Smirnov normality tests; hence, a two-tailed unpaired *t*-test was employed to identify significant differences between groups.

## RESULTS

**Fig. 2.**
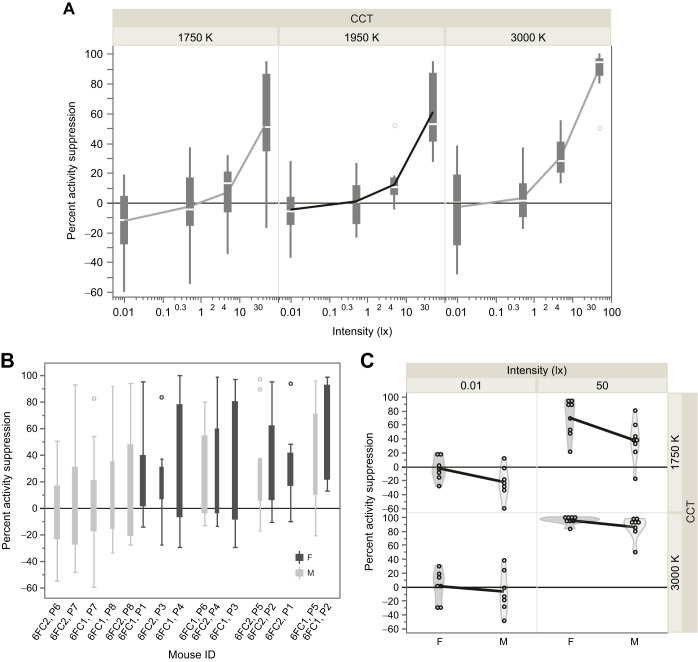
**Suppression of mouse activity by combinations of light intensity and color temperature, with illustration of individual and sex differences.** (A) Activity suppression for mice (*n*=16) by CCT and illuminance (lx). Black lines connect means, box plots indicate median, 25th and 75th percentiles, 1.5 times the interquartile range or maximum and minimum values if there are no outliers. (B) Individual differences in activity suppression for mice (*n*=16) across all combinations of illuminance and spectrum (*n*=12; 0.01–50 lx; 1750–3000 K CCT). (C) Comparison of highest and lowest combinations of spectrum (CCT=1750, 3000 K) and intensity (lx=0.01, 50) by sex (*n*=8 animals/sex). Black lines connect means.

Light intensity and color temperature affected activity patterns of mice (Fig. 2A), which had substantial variation by individual (Fig. 2B) and by sex (Fig. 2C). Light at 0.5 lx and brighter, except for the 0.5 lx/1750 K treatment, reduced activity of mice (Table 1). All 0.01 lx treatments, and the 0.5 lx/1750 K treatment, increased activity during the treatment. Within color temperatures, the effect of illuminance on mouse activity was only significantly greater for 50 lx at 1750 K, and at 0.01 lx, CCT was not statistically significant, although 1750 K CCT increased activity more than 3000 K CCT.

**Table 1.**
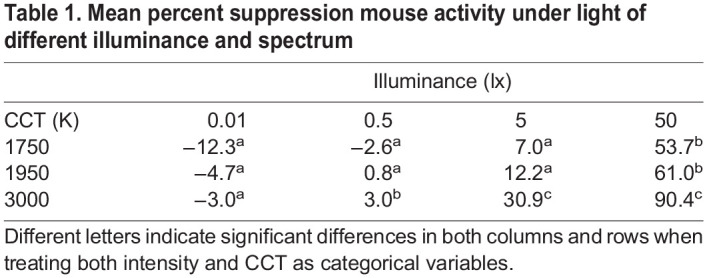
Mean percent suppression mouse activity under light of different illuminance and spectrum

Mouse activity was associated with light conditions in a generalized linear model (*P*<0.001), with significant contributions of illuminance (*P*<0.0001), spectrum (*P*<0.0001) and the interaction term (*P*<0.002). The influence of illuminance on mouse activity across the 0.01–50 lx range was several times greater than the effect of CCT across the 1750–3000 K range, with the influence of CCT decreasing at the lowest light strength.

Both sex and mouse individual added explanatory power to the model, with the model including individuals having the lowest AICc (1608.1), compared with sex (1624.6) or without either variable (1640.3). From the model including sex, activity of males was suppressed significantly less than females by light at night (*P*<0.0001; Fig. 2C). For example, at the lowest intensity (0.01 lx) and CCT, male activity was increased by 9.3% (95% CI=3.8–14.7% increase), whereas female activity was suppressed by 3.4% (95% CI=2.1% increase to 8.9% decrease). At the maximum intensity and CCT, male activity was suppressed by 85.7% (95% CI=75.6–95.7%), whereas female activity was suppressed by 98.3% (95% CI=88.3–108.3%). The model that included the animal identification number (Fig. 2B) revealed a great variability in activity among the mice, with individual mice ranging from being much more active regardless of light treatment (up to 27.9 percentage points less suppression; 95% CI=18.3–37.4) to being substantially more affected (21.5 percentage points increased suppression; 95% CI=8.1–34.8).

**Fig. 3.**
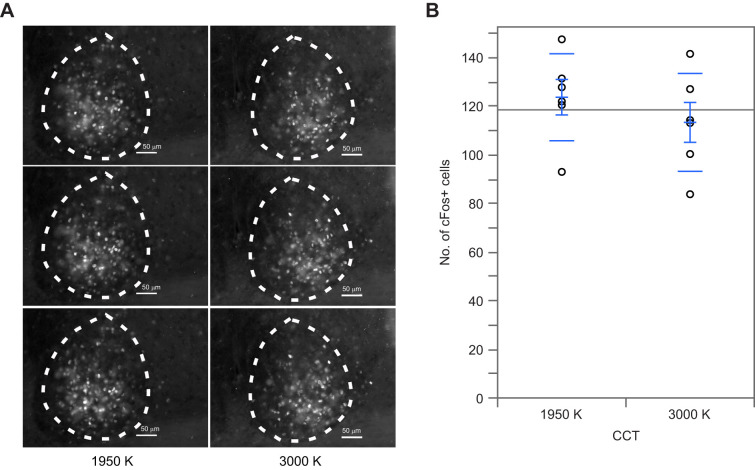
**No difference in cFos-induction in the suprachiasmatic nucleus (SCN) of wild-type mice exposed to two different CCT lights.** Mice held in 12 h:12 h light dark conditions were exposed to either a 1950 or 3000 K light (50 lx) for 1 h at ZT14, then euthanized and perfused at ZT15. (A) Representative serial images of light-evoked cFos expression in the SCN. (B) The number of immune-positive cells in both the left and right SCN from three to five consecutive coronal sections per animal were averaged to obtain one number per animal. An equal number of male and female mice was used. Results are shown as the mean±s.d. of six mice per experimental condition and were analyzed using an unpaired *t*-test (*t*_10_=0.9409, *P*<0.3689). Degrees of freedom are reported within parentheses; alpha=0.05.

The effect of lights of differing CCT (1950 and 3000 K) was tested on the light-evoked cFos response in the SCN, a classic test of the ipRGC input to the circadian system in mammals (Fig. 3). These cells express the photopigment melanopsin maximally sensitive to higher CCT (shorter wavelengths) lights (Hattar et al., 2002; Panda et al., 2005). No differences were observed in the number of cFos positive cells in the SCN of WT mice exposed to either 1950 K (123.7±17.89 s.d.) or 3000 K (113.4±8.22 s.d.) light (unpaired *t*-test: *t*_10_=0.9409, *P*<0.3689).

## DISCUSSION

We found, consistent with predictions and previous literature, that a lower CCT reduced the effect of equal intensity light (as perceived by humans) on an organism more sensitive to shorter wavelengths of light than humans when the intensity is measured in lux. The difference can be attributed to the reduced sensitivity of the rodent visual system to light in the red region of the spectrum when compared with humans. Rats and mice have sensitive low-light vision with a preponderance of rods with peak absorption at 498 nm (Bridges, 1959; Govardovskii et al., 2000), and are dichromatic, with an ultraviolet and a green cone (Jacobs et al., 2004). Although red light can be absorbed through the rod-dominated retina of rodents to form images (Nikbakht and Diamond, 2021), rodents are much less sensitive to long wavelength light, and therefore also to low CCT light than light predominated by shorter wavelengths.

Increased activity under 0.01 lx light, which is somewhat below the illumination produced by a half moon in normal conditions, is a phenomenon newly summarized in the circadian biology literature (Walbeek et al., 2021) and familiar to ecologists (Aulsebrook et al., 2022; Prugh and Golden, 2014). Many small mammal prey species reduce their activity during full moon conditions and forage more and more boldly under half moon and darker conditions (Prugh and Golden, 2014). By the same token, near-complete darkness, or infrared-dominated conditions as found in laboratory conditions with activity monitoring devices, would be darker than natural conditions and result in reduced visual acuity. Addition of light could then aid foraging behavior at relative mouse-perceived intensities and then suppress activity as the illuminance increased to levels instinctively associated with greater predation risk (e.g. 0.5 lx) and even more so as the mechanistic pathway of melatonin suppression and circadian entrainment is triggered. Moonlight entrains circadian rhythms in hamsters and mice (Butler et al., 2012; Evans et al., 2007), so this effect should be expected and increase with intensity.

A significant effect of sex as well as high variability among individuals were observed in the mouse response to different CCTs and illumination conditions. Large intraspecific variation is similarly found in the effects of light at night on other circadian processes (Phillips et al., 2019). For example, high variability was documented among individual mountain lions in response to nearby lights in a large radio-tracking study (Barrientos et al., 2023). In natural populations of wild species, individual variability is an important consideration for tolerating human-dominated landscapes. Differences in tolerance of light at night by sex, as we documented, are similarly important considerations for wild species and their conservation (Merrick and Koprowski, 2017). Furthermore, domestic mouse types have different behavioral patterns than wild mice, which would affect the interpretation of results. Comparison of relevant behaviors related to exploration and risk avoidance indicate that domestic lab mice are less wary than their wild-type conspecifics, holding true for both male (Augustsson and Meyerson, 2004) and female (Augustsson et al., 2005) mice. Accordingly, the thresholds for suppression of activity in response to light exposure in this study may be higher than would be found for wild mice.

The masking behavioral response was highly sensitive to the 3000 K light, whereas light induction of cFos within the SCN did not show any difference between exposure to equal illuminations of 1950 and 3000 K lamps. The circuit controlling this behavior is thought to go through the SCN and onto motor circuits. Hence, the simplest interpretation is that the difference is at the level of motor circuits. The findings make it unlikely that the difference in behavior is simply due to differences in the detection of the light signal. The cell populations that underlie the visual perception of brightness are normally considered to be cortical (Mazade et al., 2022), but masking behavior is not considered to involve the cerebral cortex. We expect that ipRGC and melanopsin mediate the effects of photic stimulation in the SCN, although the values reported here are lower than those usually obtained with cool white light (about 5000–6000 K). The conclusion that we can make with the obtained evidence is that the lack of difference in the number of cFos-positive cells could be because the spectral distributions associated with the two CCTs are too similar to elicit a different response, or perhaps the behavioral response is just more sensitive than the light-evoked cFos response.

Overall, these results offer important new information for the approach of mitigating the adverse effects of light at night on wildlife. Adjusting the spectrum of light to be most visible for humans while being less stimulating for other species is seen as a means to balance human needs with potential adverse impacts (Longcore, 2023; Longcore et al., 2015, 2018; Poot et al., 2008; van Grunsven et al., 2014; Wang et al., 2023). However, spectral tuning has been approached with some skepticism because it may not work across all taxa, especially those bioluminescent organisms that exploit the longer wavelengths generally used to mitigate overall effects of light at night (Owens and Lewis, 2018, 2021). Our work illustrates that the effect of intensity is much greater than the range of spectral configurations we tested, and at low enough intensities, the spectrum of the light becomes less relevant to an example behavior in a study organism. However, spectrum becomes increasingly important as intensity increases, as shown by the significant interaction term between CCT and intensity. These results suggest that when light intensity cannot be reduced (e.g. to meet design standards on a roadway), then achieving those standards with lights that overlap more with human vision than with the visual capabilities of sensitive organisms surrounding the location would be a valid mitigation approach.

A limitation of the present study is the use of a strain of mice known not to secrete melatonin although it does express its receptors (Ebihara et al., 1986). Nevertheless, the details of the mammalian circadian system from light detection to the physiological and molecular underlying mechanisms have been exhaustively characterized in this line. Hence, we chose the model with the most background information for the present work; still, future work should extend these experiments to a mouse line such as the C3H that does produce melatonin.
